# Prediction of Sphingosine protein-coding regions with a self adaptive spectral rotation method

**DOI:** 10.1371/journal.pone.0214442

**Published:** 2019-04-03

**Authors:** Zhongwei Li, Yanan Guan, Xiang Yuan, Pan Zheng, Hu Zhu

**Affiliations:** 1 College of Computer and Communication Engineering, China University of Petroleum, Qingdao, Shandong, China; 2 Department of Accounting and Information Systems, University of Canterbury, Christchurch, New Zealand; 3 College of Chemistry and Materials, Fujian Normal University, Fuzhou, China; Polytechnical Universidad de Madrid, SPAIN

## Abstract

Identifying protein coding regions in DNA sequences by computational methods is an active research topic. Welan gum produced by Sphingomonas sp. WG has great application potential in oil recovery and concrete construction industry. Predicting the coding regions in the Sphingomonas sp. WG genome and addressing the mechanism underlying the explanation for the synthesis of Welan gum metabolism is an important issue at present. In this study, we apply a self adaptive spectral rotation (SASR, for short) method, which is based on the investigation of the Triplet Periodicity property, to predict the coding regions of the whole-genome data of Sphingomonas sp. WG without any previous training process, and 1115 suspected gene fragments are obtained. Suspected gene fragments are subjected to a similarity search against the non-redundant protein sequences (nr) database of NCBI with blastx, and 762 suspected gene fragments have been labeled as genes in the nr database.

## Introduction

Genetic information is a set of general instructions that directs the translation from DNA to proteins. The vast majority of life on the earth stores genetic information in DNA sequences (some viruses store genetic information in RNA sequences). The information carried by DNA is expressed as proteins to construct cell components and perform genetic instructions for life [1]. Gene is a nucleotide sequence that can encode a substance with a certain biological function, which is the main carrier of the genetic inheritance of biological traits carrying protein information. The coding sequences of eukaryotic genes are not continuously arranged on the DNA molecule but are separated by non-coding introns, and the synthesis of protein is guided by the coding exons. Therefore, after a given genomic sequence, it is one of the central issues in bioinformatics to correctly identify the range of protein coding region in the DNA sequence and the precise position in the genomic sequence [2, 3].

Training the parameters of the biological signal model with a training set of known gene structures is an effective method for gene prediction. In general, computational methods for gene prediction can be generally categorized into three classes: (1)similarity-based prediction methods, (2) ab into techniques based on statistical models, and (3) machine learning-based methods [4–8]. For (1) and (2), various algorithms, including the Dynamic Programming (DP) or the Hidden Markov Model (HMM), are utilized to process the information gathered from these methods and subsequently predict the potential coding region of the genomic sequence [9]. Merging biological knowledge with computational techniques, machine learning aims to build a predictive model by learning the difference between coded and non-coded regions and use the learned model to predict the coding regions in the DNA sequence. The Hidden Markov Model (HMM) is the foundation of many current gene recognition algorithms [10–14]. The Hidden Markov Model considers the DNA sequence as a random process and automatically finds its internal hidden rules based on the difference in the frequency of nucleotide selection between the encoded and non-encoded DNA sequences. There are many gene identification softwares developed based on the HMM model, for example, Augustus, GeneMark.hmm [15], GENSCAN [16–20], GENIE [21], TWINSCAN [22], DOUBLESCAN [23] and Glimmer [24–28]. Among them, Augustus which is one of the most accurate gene prediction programs, adopted the Viterbi algorithm to define a probability distribution in the DNA sequence and gene structure [29–31]. After training the model with the appropriate training set, they predict the coding area with extremely high accuracy. However, training models that depend on known sequences greatly limit the adaptability of these methods, especially for new sequences from unknown organisms with no or small training sets. Therefore, in the absence of available additional information (training set), it is essential to develop some new methods to predict the locations of coding regions without any training process.

Deoxyribonucleotide (DNA) is a chain structure arranged in a certain order by four bases of A, G, T, and C. Protein is composed of polypeptide chains that are formed by 20 different amino acids. Encoding an amino acid in a protein, the genetic code requires reading 3 bases of the gene. Because of this coding relationship, the triplet of bases is called a codon. It is noted that, in most species, synonymous codons are used with different frequencies (known as codon bias) and the order with which codons are used for one protein is far from random, which raises a universal property in coding regions [32–34], called the “Triplet Periodicity (TP)”. The TP property of genes is considered to be a simple and universal distinction between coding and non-encoding regions, and studying the application of it may be an effective method to predict gene coding regions and solve other genetic problems [35–39].

In 1982, Fickett first proposed the TP property [40]. After his work, various theoretical tools were applied by researchers to investigate the TP property, such as the hidden Markov chains [41–44], the Fourier transform [17, 18], Neural Networks [45, 46], the information theory [32, 35], the time series [47–50]. Based on the Fourier transform, a method called spectral content measurement (SCM) was developed to study the TP property by Tiwari et al [18]. Since researchers have expanded and improved Tiwari’s original method in many ways, a series of methods have been developed from the original SCM [51–54]. By optimizing four coefficients in the sequence mapping and the short time Fourier transform, Anastassiou [55] proposed the optimized spectral content measure (OSCM) to compute TP property of genes. Based on the information derived from the magnitude of the discrete Fourier transform, Kotlar and Lavner [54] proposed a spectral rotation measurement (SRM) method that uses the information of the phase component such as the phase angles’ expected values and variances, to maximize the differentiation between protein coding and non-coding regions. Marhon and Sajid [56] proposed a spectrum-based technique that uses a dynamic representation scheme to map DNA sequences into a numerical form. And a post-processing method was proposed to detect the period-3 peaks instead of an empirical threshold value.

However, most high-performance methods based on TP require known genetic data from the target organism or homologous sequence data for training. Relying on training, such as the HMM-based methods, restricts the application of methods on unknown organisms, which can not offer known genetic data and homologous biological data. In addition, most of SCM related methods employ the moving slide window to investigate the TP properties for local sections of the gene sequence, and then find potential coding regions in the sequence [40, 42]. The sensitivity of these methods is highly dependent on the size of the sliding window. So, how to choose the appropriate size of the moving slide window is also one of the key issues in determining accuracy.

The research object of our work is the whole-genome sequences data of Sphingomonas sp. WG with independent intellectual property rights. Due to the lack of known genetic data and homologous biological data, the prediction methods depending on the training process cannot be employed for the whole-genome sequences data of Sphingomonas sp. WG. Chen et al. proposed a method called Adaptive Spectral Rotation (SASR) [57], which is based on the TP properties of the coding region to visualize the DNA sequence without any training process and extra information, realizing the prediction of the gene coding region. When there is insufficient training set or no extra information available, the SASR method is helpful for identifying protein coding regions on unacquainted DNA sequences. Therefore, in our work, we used the SASR method to visualize the coding region of Sphingomonas sp. WG’s whole-genome data without any training process, and the code of the SASR method is obtained from Chen’s paper.

Suspected gene fragments are obtained by manually distinguishing the position of the coding region. With comparing suspected gene fragments with the known gene in the NCBI database, we can make judgments about which the suspected gene fragments have been labeled as genes in the NCBI database. Besides, there is a high probability that real genes newly discovered in the whole-genome sequences data of Sphingomonas sp. WG will be found in the unlabeled suspected gene fragments.

## Materials and methods

### The Sphingomonas sp. WG’s whole genome data

Welan gum is an extracellular polysaccharide produced by aerobic fermentation of Sphingomonas sp [58]. With interesting rheological properties, Welan gum has been widely used as a stabilizing, suspending, emulsifying, and thickening agent in several areas such as coating materials, food, medicine, concrete additives, and enhanced oil recovery [59, 60]. Welan gum has become the hot spot of science research. In the petroleum fields, Welan gum is a new type of biological oil-displacing agent, which is of great value in tertiary oil recovery in the oil fields [61, 62]. With the deepening of the research on biochemical properties of Welan gum, its industrial value has been continuously developed. In the field of oil and natural gas extraction, Welan gum has shown great market value as an excellent tertiary oil-displacement agent.

In order to increase the yield of Welan gum and create more commercial profits, it is necessary to analyze the whole-genome sequences data of Sphingomonas sp. WG, a producer of Welan gum, and explore the biological mechanism of Welan gum synthetic route. The Bioengineering and Technology Center, Chinese University of Petroleum (East China), screened a strain of Sphingomonas sp. WG, a high-yielding strain of Welan gum, from the sea mud of Jiaozhou Bay, Qingdao. The whole-genome sequences data of Sphingomonas sp. WG was obtained by whole genome sequencing. The genomic data contains 31 scaffold sequences, 4,042,223 base pairs (bps), and the GC content of 65.88%.

### SASR

The TP profile of the base sequence is represented by a triple periodic matrix (TPM), which presented by Frenkel and Korotkov [32, 33]. The TPM is a 3 × 4-sized matrix, each row *i* (*i* = 1, 2, 3, 4) stands for a nucleotide base # (# = A, T, C or G), each column stands for a position *j* (*j* = 1, 2, 3) in the period and the entry *m*_*ij*_ is the count by which the base *i* appears at the position *j*. In the SASR, for a certain base sequence X = {*x*_*t*_ | *t* = 1, 2, 3, …, *N*}, the posterior subsequence of base sequence X at position *t*_0_ is expressed as *P*_*X*_(*t*_0_) = {*x*_*t*_ | *t*_0_ < *t* ≤ *N*}. The TP sequence, converted from the base sequence X, is represented by a sequence of TP vectors S(X) = {*s*_*t*_ | *t* = 1, 2, 3, …, *N*}, and TP verctor $s_{t}=M_{x_{t}}(P_{X}\text{(}t\text{))}$. That is, for each position *t*, the TP vector for each base # of the posterior subsequence *P*_*X*_(*t*) of the current position is calculated, ie, *M*_*A*_(*P*_*X*_(t)), *M*_*T*_(*P*_*X*_(t)), *M*_*C*_(*P*_*X*_(t)) and *M*_*G*_(*P*_*X*_(t)), and *s*_*t*_ is selected from them, according to the base at the position *t*. The TPM of the posterior subsequence at each position *t* is calculated by recursively computing M #(*P*_*X*_(*t*)) from M #(*P*_*X*_(*t* + 1)), with the initial value and the recurrence formula:

$$\begin{array}{ccc} & & M\#(P_{X}\left(N\right))=\left(0,0,0\right) \end{array}$$ (1)

$$\begin{array}{ccc} & & M\#\left(P_{X}\left(t\right)\right)=\{\begin{array}{ccc} M\#(P_{X}\left(t+1\right))>>1 & , & X_{t+1}\neq \#\\ M\#(P_{X}\left(t+1\right))>>1+\left\{1,0,0\right\} & , & X_{t+1}=\# \end{array} \end{array}$$ (2)

Remarkably, *P*_*X*_(*t* + 1) is the sequence of the posterior subsequence of *P*_*X*_(*t*). The operation “V >> n” means that the right circular shift operation is performed *n* times on the triplet row vector V.

$$\left\{Z_{1},Z_{2},Z_{3}\}\Rightarrow \{Z_{3},Z_{1},Z_{2}\right\}$$

The TP walk is a movement trajectory in the complex plane generated from the TP sequence. The moving trajectory is represented by the sequence W = {*W*_*t*_ | *t* = 0, 1, 2, …, *N*}, with the initial value *W*_0_ = {0, 0, 0}, and for each step t>0:

$$\begin{array}{ccc} & & W_{t}=\{\begin{array}{ccc} W_{t-1}+\frac{L(s_{t})}{|L(s_{t})|} & , & |s_{t}|\neq 0\\ W_{t-1} & , & |s_{t}|=0 \end{array} \end{array}$$ (3)

The function L(*x*_*t*_) maps the vector *s*_*t*_ = {*Z*_1_, *Z*_2_, *Z*_3_} into a complex number by:

$$\begin{array}{ccc} & & L\left(s_{t}\right)=Z_{1}\cdot e^{-i\frac{2\pi }{3}}+Z_{2}\cdot e^{-i\frac{4\pi }{3}}+Z_{3} \end{array}$$ (4)

The process of converting a DNA sequence into a TP vector and then generating a TP walk is called a SASR process. The recurrence equation means that, for each step *t*, the unit length is moved toward the corresponding complex number of the TP vector, in the complex plane. Therefore, TP walk can provide a good visualization of the TP properties in the complex plane. For the coding region (the region with the TP property), TP walk shows clearly and certain movement trends, and for the non-coding area, TP walk moves randomly around the stable points with insignificant movement trends. Stated thus, the difference in the visualization of the TP properties can be exploited as a basis for distinguishing the coding area from the non-coding area.

## Data experiments

### Verify the reliability and validity of the SASR method

**Fig 1:**
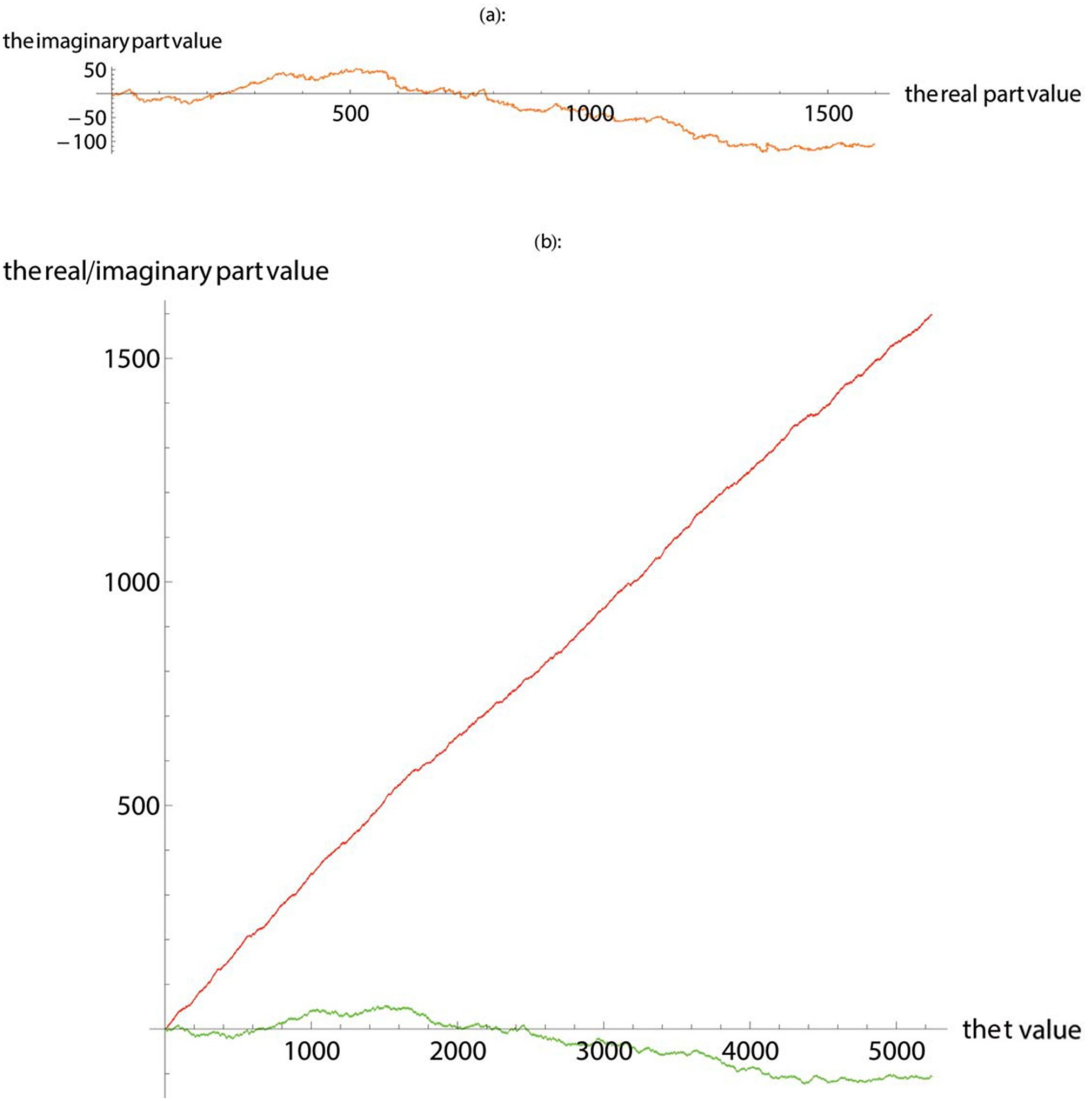
The TP walk of the coding region downloaded from the UniProt database. (**a**) Plot the walk trace in the complex plane. (**b**) Plot the real part (red) and imaginary part (green).

**Fig 2:**
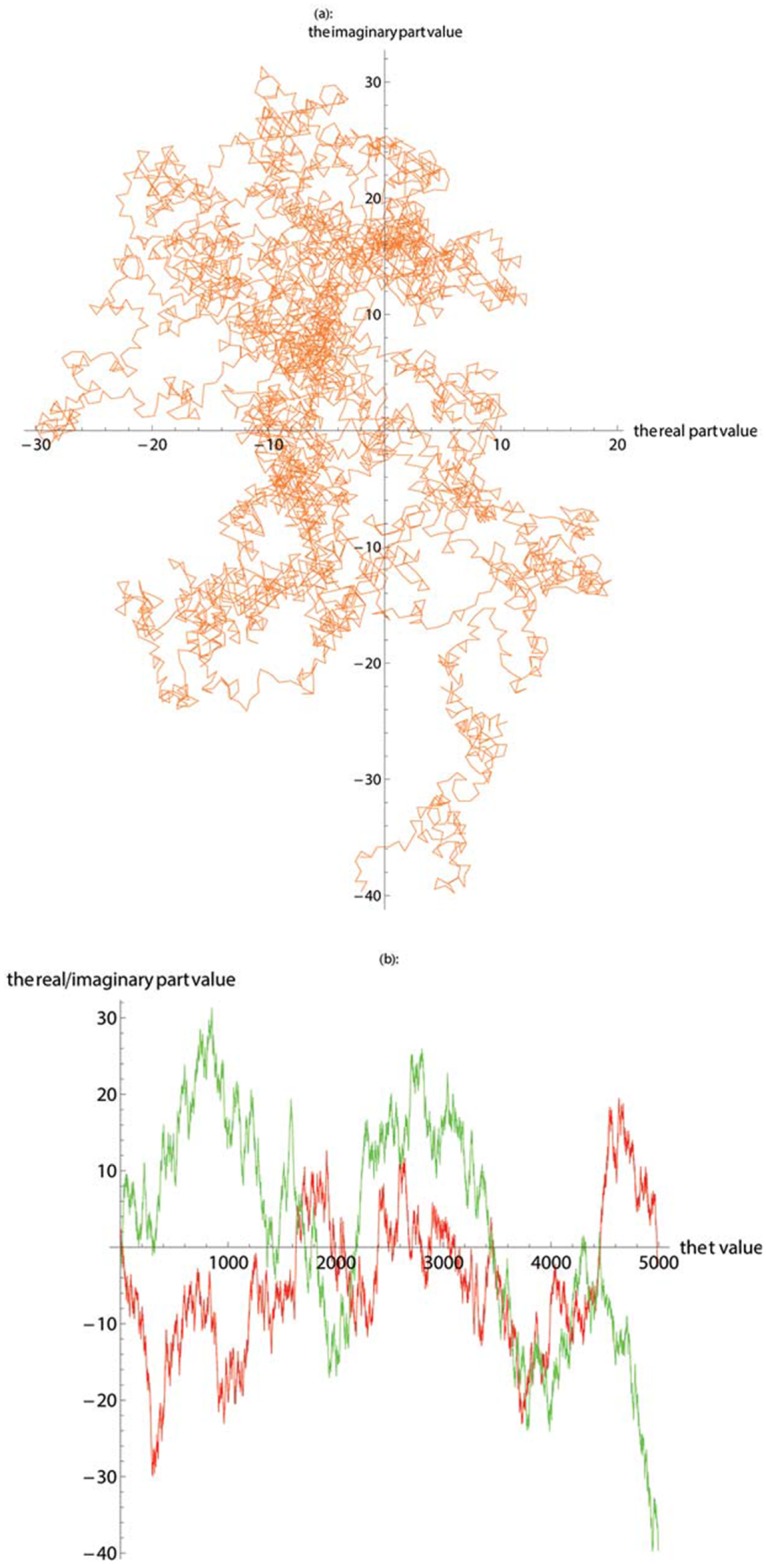
The TP walk of the randomly generated base sequence. (**a**) Plot the walk trace in the complex plane. (**b**) Plot the real part (red) and imaginary part (green).

In this work, we first apply SASR to the known gene coding regions and non-coding regions to verify the reliability and validity of the SASR method. Fig 1 shows the TP walk result of the coding region (No. J8VWM6), which encodes the proteins of Sphingomonas sp. LH128 partial outer membrane autotransporter barrel. The gene data is downloaded from the UniProt database and the length of it is 5244 bps. Fig 2 shows the TP walk result of an artificial DNA sequence generated randomly, whose length is 5000 bps. In Fig 1(**a**), TP walk moves 5244 steps in the complex plane, moving rightward from zero (0, 0) to around (1600, -100), but in Fig 2(**a**), after TP Walk moves 5000 steps in the complex plane, the walking result is randomly distributed near the zero with no specific direction. In Fig 1(**b**), the real part value keeps increasing with the increase of the *t* value, and the imaginary part remains relatively constant. However, in Fig 2(**b**), the real part and the imaginary part change more freely, and there is no fixed change pattern.

**Fig 3:**
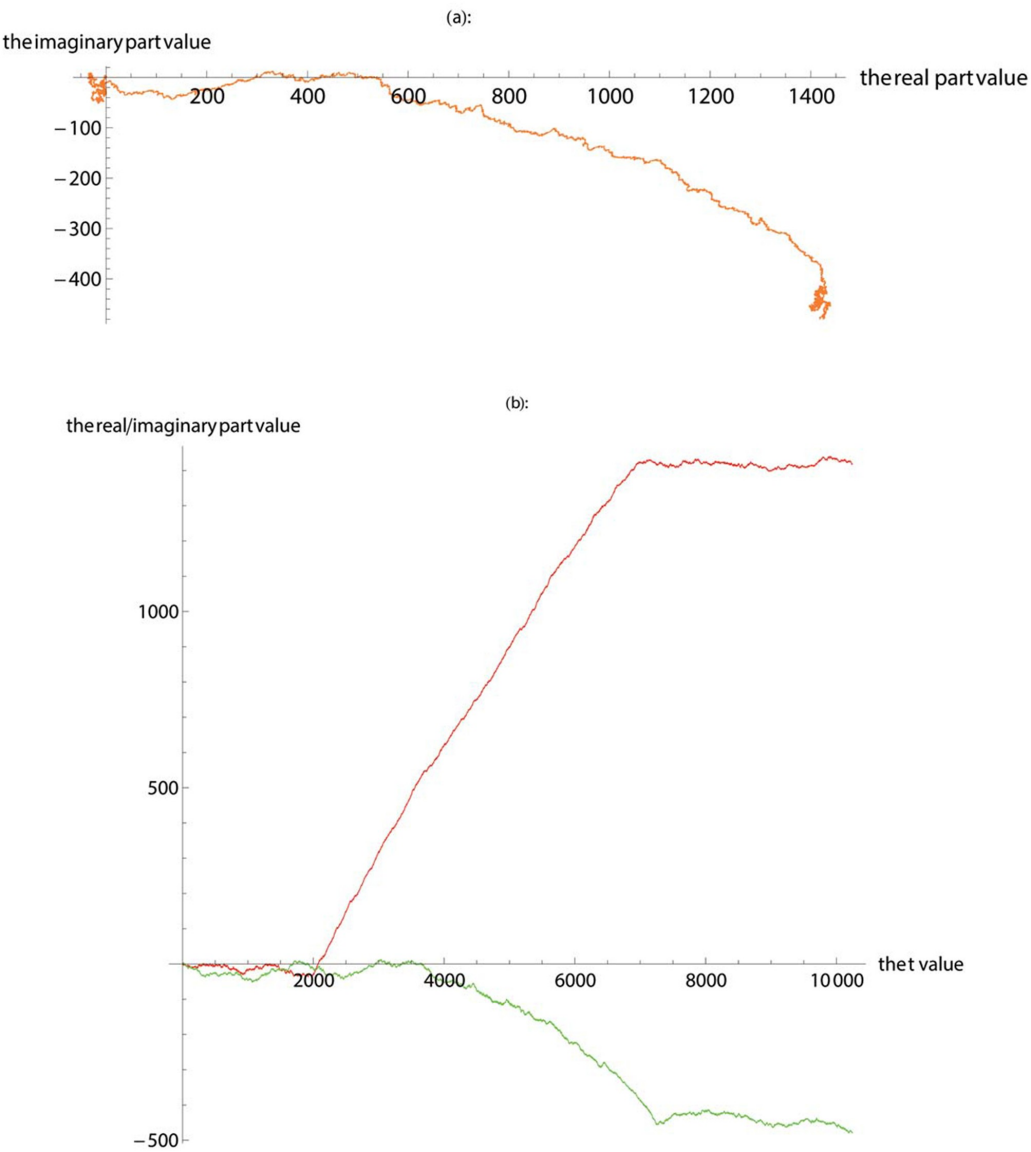
The TP walk of the new base sequence that includes both a coding region and non-coding regions. (**a**) Plot the walk trace in the complex plane. (**b**) Plot the real part (red) and imaginary part (green).

To further verify the validity of SASR, the Sphingomonas gene in the above is inserted into a randomly generated artificial DNA sequence, with the insertion position starting from 2000 to 7243, to generate a new base sequence that includes both a coding region and non-coding regions. Then using the SASR method, we get the visualization of the TP properties of the base sequence (Fig 3) in the complex plane.

In Fig 3(**a**), in the vicinity of the zero point and the end of the walk (the position of the artificial DNA sequence), the TP walk moves randomly with indefinite direction of movement, however, in the middle part (the position of inserted Sphingomonas gene), the TP walk gradually moves to the right. Meanwhile, in Fig 3(**b**), it is obvious that roughly from 2000 to 7000, with the growth of the *t* value, the real part is gradually increasing, while the artificial DNA sequence part has a smaller change in the real part value. Similar observations have been obtained after applying the SASR method to other known coding regions and non-coding regions. The reliability and validity of the SASR method have been verified through the above experiments. It can be seen that there is a large difference between the coding and non-coding regions in the graphic output of TP Walk. Therefore, after applying the SASR method to the base sequence being measured, by observing the change of the TP Walk’s real part and referring to the trend of TP walking in the complex plane, the coding and non-coding areas can be distinguished without any training process.

### Predicting the protein-coding regions of the Sphingomonas sp. WG’s whole genome data

**Fig 4:**
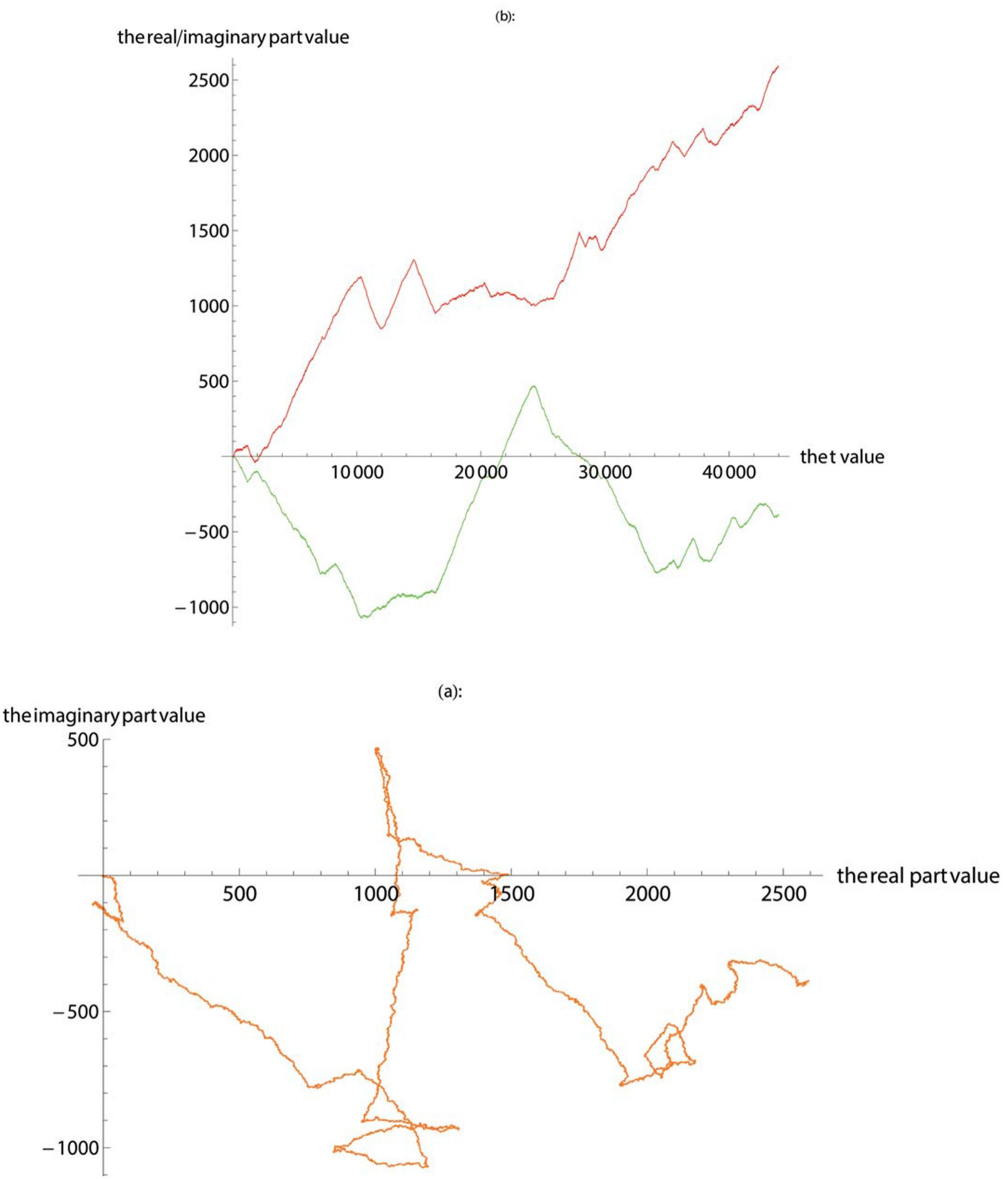
The TP walk of scaffold 21. (**a**) Plot the walk trace in the complex plane. (**b**) Plot the real part (red) and imaginary part (green).

The SASR method is applied to the 31 scaffolds of the Sphingomonas sp. WG’s whole-genome data respectively to predict probable coding regions. Here, taking the processing of No. 21 scaffold as an example to illustrate the prediction processing of the possible coding regions. First, the SASR method is applied to No. 21 scaffold (the sequence length is 43986), and the visualization of the TP properties is obtained (Fig 4).

According to the difference in the graphic output between the coding and non-coding regions, the fragment of the base sequence that corresponds to the characteristics of the coding region is identified, which is called a suspected gene fragment, and the specific position of the fragment is identified. Since one base sequence may contain multiple segments of coding regions, a plurality of base segments that conform to the characteristics of the coding region can be found in Fig 4, such as 2000-7000, 16000-20000 and so on. Because the number of bases is large, the trend of the real part of the partial TP walk may not be obvious. So, it is possible to cut out the invisible part of the fragment and use the SASR method again to obtain the position of the base fragment that corresponds to the coding region. Based on the position of the suspected gene fragment in No. 21 scaffold, the base sequence is divided using Matlab software to obtain multiple suspected gene fragments. Finally, the obtained suspected gene fragments are compared with the known gene in the NCBI nr database with blastx.

## Results

**Fig 5:**
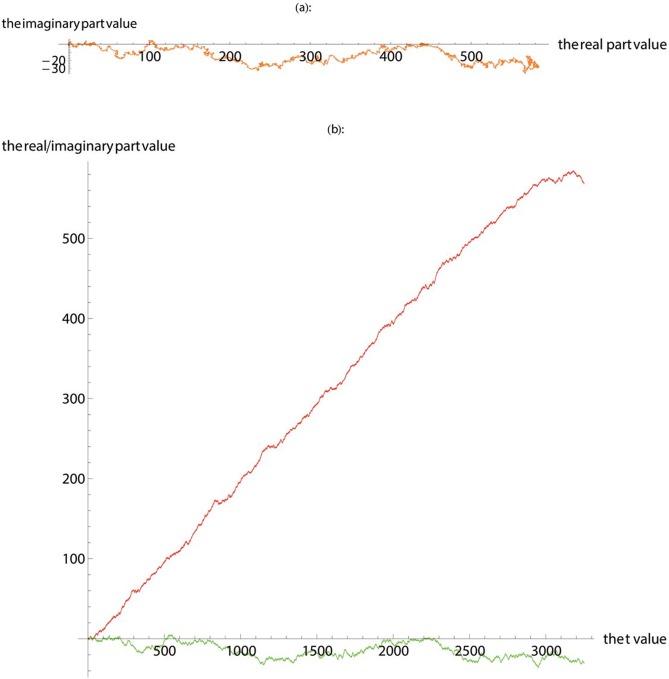
The TP walk of the suspected gene fragment 21-21000-24250. (**a**) Plot the walk trace in the complex plane. (**b**) Plot the real part (red) and imaginary part (green).

**Fig 6:**
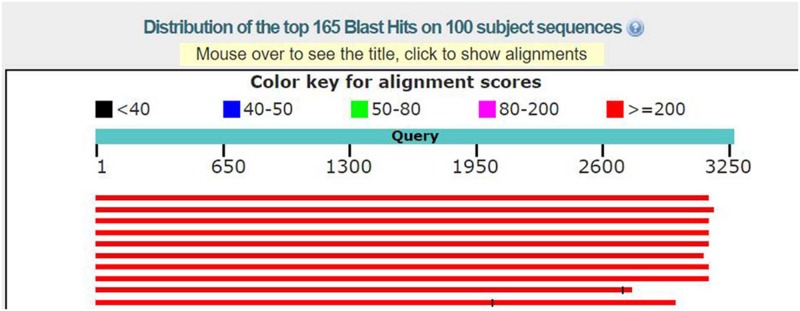
Graphic summary of the sequence alignment result of the suspected gene fragment 21-21000-24250. (a) Similarity color diagram, in which the similarity is arranged from high to low: red, purple, green, blue, black. (b) The suspected gene fragment has the highest degree of similarity and the best matching degree to the first gene sequence.

The graphic output of the base sequence fragment, whose position starting from 21000 to 24250 (Simply expressed as 21-21000-24250, and other suspected gene fragments are expressed in the same way) in No. 21 scaffold, meets the characteristics of the coding region (Fig 5). The suspected gene fragment is compared with the known gene in the NCBI nr database using Blast software. The basic information of this sequence alignment please refer to the Table 1, such as the type of molecule, the length of the sequence, the name of the database to be compared, and the program used, etc. And the results of sequence alignment are shown in Fig 6 and Table 2.

**Table 1:** The basic information of the comparison result of the suspected gene fragment 21-21000-24250.

Item	Content
RID	SETZNMME01R
Query ID	lcl—Query_217685
Description	None
Database Name	nr
Description	All non-redundant GenBank CDS translations+ PDB+ SwissProt+ PIR+ PRF excluding environmental samples from WGS projects
Program	BLASTX 2.8.0+
Molecule type	nucleic acid
Query Length	3251

**Table 2:** The first 8 detailed alignment results of the suspected gene fragment 21-21000-24250 and different sequences in the database. Five metrics are used to evaluate the match results of the query sequence: Max score, Total score, Query coverage, E value, and Ident.

Description	Max score	Total score	Query cover	E value	Ident
MULTISPECIES: hybrid sensor histidine kinase/response regulator [Sphingomonas]	1927	1927	96%	0.0	100%
PAS domain S-box protein [Sphingomonas pituitosa]	1570	1570	96%	0.0	81%
PAS domain S-box protein [Sphingomonas spermidinifaciens]	1523	1523	96%	0.0	79%
PAS domain S-box-containing protein [Sphingomonas jatrophae]	1269	1269	96%	0.0	66%
PAS domain-containing sensor histidine kinase [Sphingomonas jatrophae]	1052	1259	83%	0.0	66%
PAS domain S-box protein [Phenylobacterium deserti]	812	1118	90%	0.0	69%
PAS domain S-box protein [Asticcacaulis sp. CF398]	744	1028	84%	0.0	51%
PAS domain S-box protein [Methylobacterium platani]	699	941	94%	0.0	55%

In Fig 6, the color keys of different colors represent the magnitude of similarity. The more red regions, the higher the similarity of the fragments that are matched in the database. Simultaneously, the matched gene sequences are ranked by the matching scores from large to small, so the suspected gene fragment has the highest degree of similarity and the best matching degree to the first gene sequence. In Table 2, two of the most noteworthy values are the ident value and the E value. The ident value indicates the degree of similarity between the aligned sequence and the target sequence, that is, the number of bases on the match as a percentage of the total sequence length. The E value indicates the possibility of random matching. The greater the E value, the greater the likelihood of random matching. When the E value is close to zero or zero, it can be considered as an exact match. Table 2 shows, the sequence alignment results of the suspected gene fragment 21-21000-24250 and the alignment of hybrid sensor histidine kinase/response regulator [Sphingomonas] is the best one, whose ident value is 100% and the E value is 0, which can be considered as an exact match.

To further verify the reliability of the sequence alignment results, the PSI-BLAST program was used to search for similar sequences of the suspected gene fragments 21-21000-24250 by multiple iterations in the database (the threshold is 0.001). There are several highly similar protein sequences in the summary table (hits reported in the 1^st iteration with E value is 0.0 and sequence identity > 30%; see Table 3 for details). The newly added sequences that were below the threshold in the previous search are indicated as “new” and “old” indicates that the sequence has been searched before this iteration. Table 3 contains the sequence alignment information of the first 5 sequences in the search results of the previous eight iterations. It can be seen that from the third iteration, although the order of the sequences in the search results is not exactly the same, the sequences with high similarity are still in front. After several iterations, the searched new sequence is gradually reduced, and when no new sequences are detected below the defined threshold, the iterative process is terminated. Therefore, through multiple iterative searches of PSI-BLAST, we can find that the suspected gene fragment 21-21000-24250 has distant sequence similarity with the sequence, whose entry name is A0A1A3QEF8_MYCSZ in UniProtKB, and can speculate on the possible structure and function of the protein compiled by the suspected gene fragment.

**Table 3:** The PSI-BLAST search results of the suspected gene sequence 21-21000-24250. It contains the sequence alignment information of the first 5 sequences in the search results of the previous eight iterations. The similar sequences in search results sort descending based on score values.

iteration	Alignment	DB:ID	Length	Score(Bits)	Identities	Positives	E()
1^st	1 new	TR:W4ZEQ5_STRPU	7246	1156.0	32.0	32.0	0.0
	2 new	TR:W4YYP3_STRPU	11309	1053.0	28.0	33.0	0.0
	3 new	TR:B2HHB3_MYCMM	3634	962.0	33.0	35.0	0.0
	4 new	TR:A0A2N2JNV2_9DELT	11114	946.0	27.0	30.0	0.0
	5 new	TR:A0A0D2WHT8_CAPO3	7350	914.0	24.0	30.0	0.0
2^st	1 old	TR:B2HHB3_MYCMM	3634	2873.0	30.0	32.0	0.0
	2 old	TR:A0A1X1REZ8_9MYCO	4053	2804.0	27.0	28.0	0.0
	3 old	TR:A0A1A3KX53_MYCGO	3739	2791.0	27.0	29.0	0.0
	4 old	TR:A0A1A3QEF8_MYCSZ	4843	2784.0	27.0	29.0	0.0
	5 old	TR:A0A1X1SZH0_9MYCO	3642	2759.0	27.0	29.0	0.0
3^st	1 old	TR:A0A1A3QEF8_MYCSZ	4843	3685.0	25.0	27.0	0.0
	2 old	TR:A0A1X1REZ8_9MYCO	4053	3684.0	26.0	27.0	0.0
	3 old	TR:A0A1A3KX53_MYCGO	3739	3653.0	26.0	27.0	0.0
	4 old	TR:A0A1A3NH48_MYCAS	5961	3648.0	25.0	27.0	0.0
	5 old	TR:A0A1X1WR67_MYCGO	5961	3636.0	25.0	26.0	0.0
4^st	1 old	TR:A0A1A3QEF8_MYCSZ	4843	4076.0	25.0	27.0	0.0
	2 old	TR:A0A1X1REZ8_9MYCO	4053	4052.0	24.0	26.0	0.0
	3 old	TR:A0A1A3NH48_MYCAS	5961	4028.0	23.0	25.0	0.0
	4 old	TR:A0A1X1WR67_MYCGO	5961	4018.0	24.0	26.0	0.0
	5 old	TR:A0A1A3KPQ5_MYCAS	4945	4010.0	24.0	26.0	0.0
5^st	1 old	TR:A0A1A3QEF8_MYCSZ	4843	4188.0	24.0	25.0	0.0
	2 old	TR:A0A1X1REZ8_9MYCO	4053	4184.0	24.0	25.0	0.0
	3 old	TR:A0A1A3NH48_MYCAS	5961	4183.0	23.0	25.0	0.0
	4 old	TR:A0A1X1WR67_MYCGO	5961	4163.0	24.0	26.0	0.0
	5 old	TR:A0A0Q2QJK1_MYCGO	6569	4142.0	24.0	26.0	0.0
6^st	1 old	TR:A0A1A3QEF8_MYCSZ	4843	4205.0	22.0	23.0	0.0
	2 old	TR:A0A1A3NH48_MYCAS	5961	4197.0	23.0	25.0	0.0
	3 old	TR:A0A1X1WR67_MYCGO	5961	4234.0	23.0	25.0	0.0
	4 old	TR:A0A1A3NH48_MYCAS	5961	4226.0	24.0	25.0	0.0
	5 old	TR:A0A0Q2QJK1_MYCGO	6569	4213.0	23.0	24.0	0.0
7^st	1 old	TR:A0A1A3QEF8_MYCSZ	4843	4270.0	22.0	23.0	0.0
	2 old	TR:A0A1X1REZ8_9MYCO	4053	4240.0	23.0	24.0	0.0
	3 old	TR:A0A1X1WR67_MYCGO	5961	4181.0	23.0	25.0	0.0
	4 old	TR:A0A1X1REZ8_9MYCO	4053	4177.0	23.0	25.0	0.0
	5 old	TR:A0A0Q2QJK1_MYCGO	6569	4164.0	23.0	24.0	0.0
8^st	1 old	TR:A0A1A3QEF8_MYCSZ	4843	4293.0	22.0	24.0	0.0
	2 old	TR:A0A1X1REZ8_9MYCO	4053	4271.0	23.0	24.0	0.0
	3 old	TR:A0A1A3NH48_MYCAS	5961	4263.0	22.0	24.0	0.0
	4 old	TR:A0A1X1WR67_MYCGO	5961	4209.0	23.0	24.0	0.0
	5 old	TR:A0A0Q2QJK1_MYCGO	6569	4024.0	22.0	24.0	0.0

The above can prove that by processing the No. 21 scaffold base sequence using the SASR method, the coding region of No. 21 scaffold is found, and the coding region has been labeled as genes in the NCBI database.

After the SASR method is applied to predict the coding regions of the 31 scaffolds of the Sphingomonas sp. WG’s whole genome data, 1115 suspected gene fragments are obtained in total by slicing the base sequences. These results can, to a certain extent, prove that these suspected gene segments have been labeled as genes or there are gene sequences with high similarity to suspected gene fragments in NCBI database. So it can be considered that they are base sequences with the function of protein coding, further illustrating the reliability and validity of the SASR method.

There are 353 suspected gene fragments not matched against any known gene sequences in the NCBI database. On the basis of that the reliability and validity of the SASR method have been verified, we can consider that the 353 suspected gene fragments are newly discovered suspected gene fragments with the function of protein coding that have not been included in the NCBI database in high probability. But, whether or not the 353 suspected gene fragments are truly gene sequences with the function of protein coding, and what their corresponding biological functions are, it is necessary to do corresponding biological experiments to further verify. However, due to differences in specialized fields, the lack of relevant biological theory knowledge, biological experimental procedures, and professional equipment, and the too high cost of manpower and material resources to complete biometric verification experiments, we can not independently carry out follow-up verification against the suspected gene fragments, and it is necessary to cooperate with other specialized biological laboratories.

## Discussion

Without any preceding training process, the SASR method based on the TP property of the coding region provides a visualized presentation of unannotated protein-coding regions in DNA sequences, which implements the prediction of the coding regions in the DNA sequence. According to the visualization of the DNA sequence, the starting and ending positions of the suspected gene fragment are manually determined with certain errors. The error range can be controlled within 100 bases. Since the DNA base sequence has a large number of bases, mostly in the order of 10,000 and 100,000 digits, the magnitude of the error can reach 10^−3^ or even smaller, which can be accepted. The results of our work provide great reference value for biological experimental workers to identify protein coding regions in the whole-genome sequences data of Sphingomonas sp. WG, and it will greatly reduce their workload and improve their efficiency. In the follow-up work, we hope to cooperate with biological experimental workers to find real genes with protein-coding functions in unlabeled suspected gene fragments through biological experiments and to make gene function annotations, and further develop an efficient new algorithm that can extract the numerical results of the coding region prediction from the SASR’s graphical output, instead of manual segmentation, thereby improving the accuracy of the location of suspected gene segments.

## Supporting information

S1 Dataset DNA sequence dataset to replicate the analyses. The dataset includes: J8VWM6 (a segment of DNA is downloaded from the UniProt database and the length of it is 5244 bps); 5000test(an artificial DNA sequence generated randomly); 5000test-2000-7243 (the DNA sequence J8VWM6 is inserted into a randomly generated artificial DNA sequence, with the insertion position starting from 2000 to 7243); Scaffold21 (a segment of DNA of the Sphingomonas sp. WG’s whole-genome data); 21-21000-24250 (the base sequence fragment whose position starting from 21000 to 24250 in No. 21 scaffold).
(ZIP)

## References

[1] Michel Morange. The Central Dogma of Molecular Biology. Resonance, 2009, 14(3):236–247. 10.1007/s12045-009-0024-6

[2] Verma S, Kumar D. Detection of Protein Coding Regions using Goertzel Algorithm. Propagation characteristics of free-space terahertz electromagneticpulses. 2015.

[3] Putluri S R, Rahman M Z U. Identification of Protein Coding Region in DNA Sequence Using Novel Adaptive Exon Predictor. 2018.

[4] Yang H, Lv H, Ding H, et al iRNA-2OM: A Sequence-Based Predictor for Identifying 2’-O-Methylation Sites in Homo sapiens. Journal of Computational Biology, 2018 10.1089/cmb.2018.0004 30113871

[5] Song T., Rodriguez-Paton Alfonso, Zheng P., Zeng X., Spiking Neural P Systems With Colored Spikes, IEEE Transactions on Cognitive and Developmental Systems, 2018 10.1109/TCDS.2017.2785332 30281471

[6] Wei L, Chen H, Su R. M6APred-EL: A Sequence-Based Predictor for Identifying N6-methyladenosine Sites Using Ensemble Learning. Molecular Therapy-Nucleic Acids, 2018, 12: 635–644. 10.1016/j.omtn.2018.07.004 30081234 PMC6082921

[7] Liu G, Liu G J, Tan J X, et al DNA physical properties outperform sequence compositional information in classifying nucleosome-enriched and-depleted regions. Genomics, 2018 10.1016/j.ygeno.2018.07.013 30055231

[8] Manavalan B, Shin T H, Lee G. DHSpred: support-vector-machine-based human DNase I hypersensitive sites prediction using the optimal features selected by random forest. Oncotarget, 2018, 9(2): 1944 10.18632/oncotarget.23099 29416743 PMC5788611

[9] Do J H, Choi D K. Computational Approaches to Gene Prediction. Journal of Microbiology, 2006, 44(2):137–144. 16728949

[10] Richard Durbin. Biological Sequence Analysis: Probabilistic Models of Proteins and Nucleic Acids. Cambridge University Press, 1998:549–552.

[11] Ewan Birney. Hidden Markov Models in Biological Sequence Analysis. BM Corp., 2001,45(3.4):449–454

[12] Azad R K, Borodovsky M. Probabilistic Methods of Identifying Genes in Prokaryotic Genomes: Connections to the HMM Theory. Briefings in Bioinformatics, 2004, 5(2):118–130. 10.1093/bib/5.2.118 15260893

[13] Byung Jun Yoon. Hidden Markov Models and Their Applications in Biological Sequence Analysis. Current Genomics, 2009, 10(6):402 10.2174/138920209789177575 20190955 PMC2766791

[14] Mørk S, Holmes I. Evaluating Bacterial Gene-Finding HMM Structures as Probabilistic Logic Programs. Oxford University Press, 2012, 28(5):636. 10.1093/bioinformatics/btr698 PMC3289911 22215819

[15] Borodovsky M, Lomsadze A. Eukaryotic Gene Prediction Using GeneMark.hmm-E and GeneMark-ES. Curr Protoc Bioinformatics, 2011, Chapter 4(1):Unit 4.6.1 10.1002/0471250953.bi0406s35 PMC3204378 21901742

[16] Lukashin A V, Borodovsky M. GeneMark.hmm: New Solutions for Gene Finding. Nucleic Acids Research, 1998, 26(4):1107–1115. 10.1093/nar/26.4.1107 9461475 PMC147337

[17] Issac B, Singh H, Kaur H, et al Locating Probable Genes Using Fourier Transform Approach. Bioinformatics, 2002, 18(1):196–197. 10.1093/bioinformatics/18.1.196 11836230

[18] Song T, Zeng X., Zheng P., Jiang M., Rodriguez-Paton A., A Parallel Workflow Pattern Modelling Using Spiking Neural P Systems With Colored Spikes, IEEE Transactions on Nanobioscience, 13(3):263–270. 10.1109/TNB.2018.2873221 30281471

[19] Aggarwal G, Ramaswamy R. Ab Initio Gene Identification: Prokaryote Genome Annotation with GeneScan and GLIMMER. J Biosci, 2002, 27(1):7–14. 10.1007/BF02703679 11927773

[20] Akhtar M, Epps J, Ambikairajah E. On DNA Numerical Representations for Period-3 Based Exon Prediction. IEEE International Workshop on Genomic Signal Processing and Statistics, 2007:1–4. 10.1109/GENSIPS.2007.4365821

[21] Reese M G, Kulp D, Tammana H, et al Genie–Gene Finding in Drosophila Melanogaster. Genome Research, 2000, 10(4):529 10.1101/gr.10.4.529 10779493 PMC310881

[22] Wang M, Buhler J, Brent M R. The Effects of Evolutionary Distance on TWINSCAN, an Algorithm for Pair-Wise Comparative Gene Prediction. Cold Spring Harbor Symposia on Quantitative Biology, 2003, 68(3):125–130. 10.1101/sqb.2003.68.125 15338610

[23] Uretavidal A, Ettwiller L, Birney E. Comparative Genomics: Genome-Wide Analysis in Metazoan Eukaryotes. Nature Reviews Genetics, 2003, 4(4):251–62. 10.1038/nrg1043 12671656

[24] Delcher A L, Harmon D, Kasif S, et al Improved Microbial Gene Identification with GLIMMER. Nucleic Acids Research, 1999, 27(23):4636–41. 10.1093/nar/27.23.4636 10556321 PMC148753

[25] Roy S S, Barman S. Identification of protein coding region of DNA sequence using multirate filter Computational Advancement in Communication Circuits and Systems. Springer India, 2015.

[26] Farsani M S, Sahhaf M R A, Abootalebi V. Performance Improvement of the Goertzel Algorithm in Estimating of Protein Coding Regions Using Modified Anti-notch Filter and Linear Predictive Coding Model. Journal of Medical Signals & Sensors, 2016, 6(3):130–140. 27563569 PMC4973456

[27] Arthur S S, Delcher A L, Kasif S, et al Microbial Gene Identification Using Interpolated Markov Models. Nucleic Acids Research. 1998, 26(2):544 10.1093/nar/26.2.544 9421513 PMC147303

[28] Delcher A L, Bratke K A, Powers E C, et al Identifying Bacterial Genes and Endosymbiont DNA with Glimmer. Bioinformatics, 2007, 23(6):673–679. 10.1093/bioinformatics/btm009 17237039 PMC2387122

[29] Guda C. Bioinformatic Methods and Resources for Neuroscience Research, Current Laboratory Methods in Neuroscience Research. Springer New York, 2014. Stanke M, Steinkamp R, Waack S, et al. AUGUSTUS: a Web Server for Gene Finding in Eukaryotes. Nucleic Acids Research, 2004, 32(Web Server issue):309-12. 10.1093/nar/gkh379 PMC441517 15215400

[30] Song T., Wang X., Zhang Z., Chen Z., Homogenous Spiking Neural P Systems with Anti-spikes, Neural Computing and Applications. 2014, 24(7-8), 1833–1841 10.1007/s00521-013-1397-8

[31] Stanke M, Schöffmann O, Morgenstern B, et al Gene Prediction in Eukaryotes with a Generalized Hidden Markov Model That Uses Hints From External Sources. Bmc Bioinformatics, 2006, 7(1):62 10.1186/1471-2105-7-62 16469098 PMC1409804

[32] Frenkel F E, Korotkov E V. Classification Analysis of Triplet Periodicity in Protein-Coding Regions of Genes. Gene, 2008, 421(1):52–60. 10.1016/j.gene.2008.06.012 18593596

[33] Korotkov E V, Korotkova M A. Study of The Triplet Periodicity Phase Shifts in Genes. Journal of Integrative Bioinformatics, 2010, 7(3):219–230. 10.1515/jib-2010-131 20375465

[34] Stein L D, Wang L. Localizing Triplet Periodicity in DNA and cDNA Sequences. Bmc Bioinformatics, 2010, 11(1):1–8. 21059240 10.1186/1471-2105-11-550 PMC2992068

[35] Frenkel F E, Korotkov E V. Using Triplet Periodicity of Nucleotide Sequences for Finding Potential Reading Frame Shifts in Genes. DNA Research: An International Journal for Rapid Publication of Reports on Genes and Genomes, 2009, 16(2):105 10.1093/dnares/dsp002 19261626 PMC2671204

[36] Frenkel F E, Korotkov E V. Classification of Triplet Periodicity in The DNA Sequences of Genes From KEGG Databank. Molecular Biology, 2008, 42(4):629–640. 10.1134/S0026893308040201 18856072

[37] Saberkari H, Shamsi M, Heravi H, et al A Novel Fast Algorithm for Exon Prediction in Eukaryotic Genes Using Linear Predictive Coding Model and Goertzel Algorithm Based On The Z-Curve. International Journal of Computer Applications, 2013, 67(17):25–38.

[38] Suvorova Y M, Rudenko V M, Korotkov E V. Detection Change Points of Triplet Periodicity of Gene. Gene, 2012, 491(1):58–64. 10.1016/j.gene.2011.08.032 21982972

[39] Sharma S, Sandal K, Garg P, et al. Performance analysis of window functions for exon prediction in DNA sequences. International Conference on Computing. IEEE, 2017.

[40] Fickett J W. Recognition of Protein Coding Regions in DNA Sequences. Nucleic Acids Research, 1982, 10(17):5303–5318. 10.1093/nar/10.17.5303 7145702 PMC320873

[41] Henderson J, Salzberg S, Fasman K H. Finding Genes in DNA with a Hidden Markov Model. Journal of Computational Biology, 1997, 4(2):127–141. 10.1089/cmb.1997.4.127 9228612

[42] Kulp D, Haussler D, Reese M G, et al A Generalized Hidden Markov Model for The Recognition of Human Genes in DNA. International Conference on Intelligent Systems for Molecular Biology, 1996,4:353–142. 8877513

[43] Krogh A, Mian I S, Haussler D. A hidden Markov model that finds genes in E. coli DNA. Nucleic Acids Research, 1994, 22(22): 4768–4778. 10.1093/nar/22.22.4768 7984429 PMC308529

[44] Song T., Pan L., Wu T., Zheng P., Wong M. L. Dennis and Rodriguez-Paton A., Spiking Neural P Systems with Learning Functions, IEEE Trans Nanobioscience, 2019 10.1109/TNB.2019.2896981 30716044

[45] Snyder E E, Stormo G D. Identification of Coding Regions in Genomic DNA Sequences: an Application of Dynamic Programming and Neural Networks. Nucleic Acids Research, 2011, 21(3):607–13. 10.1093/nar/21.3.607 PMC309159 8441672

[46] Thomas A, Skolnick M H. A Probabilistic Model for Detecting Coding Regions in DNA Sequences. Ima J Math Appl Med Biol, 1994, 11(3):149–160. 10.1093/imammb/11.3.149 7822887

[47] Song T, Liu X, Zhao Y, Zhang X, Spiking Neural P Systems with White Hole Neurons, IEEE Trans on Nanobioscience, 2016, 15(7): 666–673. 10.1109/TNB.2016.2598879 28029614

[48] Cao Y, Tung W, Gao J B. Recurrence Time Statistics: Versatile Tools for Genomic DNA Sequence Analysis. Journal of Bioinformatics and Computational Biology, 2004, 3(03):-. 10.1142/S0219720005001235 16108089

[49] Song T, Pan L, Spiking Neural P Systems with Request Rules, Neurocomputing, 2016, 193(12): 193–200. 10.1016/j.neucom.2016.02.023

[50] Pang S., Ding T., Rodriguez-Paton A., Song T., Zheng P., A Parallel Bioinspired Framework for Numerical Calculations Using Enzymatic P System with an Enzymatic Environment. 10.1109/ACCESS.2018.2876364

[51] Song T., Zheng P. Wong D.M., Wang X., Design of Logic Gates Using Spiking Neural P Systems with Homogeneous Neurons and Astrocytes-like Control, Information Sciences, 2016, 372, 380–391. 10.1016/j.ins.2016.08.055

[52] Yin C, Yau S T. Prediction of Protein Coding Regions by The 3-Base Periodicity Analysis of a DNA Sequence. Journal of Theoretical Biology, 2007, 247(4):687–694. 10.1016/j.jtbi.2007.03.038 17509616

[53] Sangill R, Rastrupandersen N, Bildsoe H, et al Optimized Spectral Editing of 13 C MAS NMR Spectra of Rigid Solids Using Cross-Polarization Methods. Journal of Magnetic Resonance, 1994, 107(1):67–78. 10.1006/jmra.1994.1048

[54] Kotlar D, Lavner Y. Gene Prediction by Spectral Rotation Measure: A New Method for Identifying Protein-Coding Regions. Genome Research, 2003, 13(8):1930 10.1101/gr.1261703 12869578 PMC403785

[55] Anastassiou D. Frequency-domain Analysis of Biomolecular Sequences. Bioinformatics, 2000, 16(12):1073–1081. 10.1093/bioinformatics/16.12.1073 11159326

[56] Marhon Sajid A. Nucleotide distribution variance-based dynamic representation scheme for novel gene prediction. Network Modeling Analysis in Health Informatics and Bioinformatics, 2015, 4(1):31 10.1007/s13721-015-0103-4

[57] Chen B, Peng Ji, Numericalization of the self adaptive spectral rotation method for coding region prediction, Journal of Theoretical Biology, 2011, 296: 95–102 10.1016/j.jtbi.2011.12.002 22178641

[58] O’Neill M A, Selvendran R R, Morris V J, et al Structure of The Extracellular Polysaccharide Produced by The Bacterium Alcaligenes, (ATCC 31555) species. Carbohydrate Research, 1986, 147(2):295–313. 10.1016/S0008-6215(00)90638-4 3708628

[59] Li H, Xu H, Xu H, et al Enhanced Welan Gum Production Using a Two-Stage Agitation Speed Control Strategy in Alcaligenes sp. CGMCC2428. Bioprocess and Biosystems Engineering, 2011, 34(1):95–102. 10.1007/s00449-010-0450-6 20640447

[60] Gao Changhong. Potential of Welan Gum as mud thickener. Journal of Petroleum Exploration and Production Technology, 2015, 5(1):109–112. 10.1007/s13202-014-0114-1

[61] Gao C. Potential Applications of Welan Gum in Upstream Petroleum Industry. International Journal of Oil, Gas and Coal Engineering, 2016, 4(2): 16 10.11648/j.ogce.20160402.12

[62] Stankowski J D, Zeller S G. Location of The O-Acetyl Group in Welan by The Reductive-Cleavage Method. Carbohydr Res, 1992, 224(3):337–341. 10.1016/0008-6215(92)84122-9 1591770

